# Calibrated, cost-sensitive, explainable ensemble learning for customer churn prediction in retail banking: a leakage-safe pipeline and single-dataset case study

**DOI:** 10.3389/frai.2026.1889639

**Published:** 2026-09-17

**Authors:** Farhana Karim, Md. Ishtiaque Alam, Aram Lalafaryan, Artyom Mkrtchyan, Anzhela Hovakimyan, Md Mustafizur, Gazi Touhidul Alam, Mia Md. Tofayel Gonee Manik

**Affiliations:** 1 Harrison College of Business and Computing, Southeast Missouri State University, Cape Girardeau, MO, United States; 2 Orfalea College of Business, California Polytechnic State University, San Luis Obispo, CA, United States; 3 Department of Business Administration, Westcliff University, Irvine, CA, United States; 4 Department of Business Administration, Stanton University, Los Angeles, CA, United States; 5 Department of Business Administration, Merit University, Los Angeles, CA, United States; 6 Department of Business Administration, University of the Potomac, Washington, DC, United States; 7 College of Graduate and Professional Studies, Trine University, Detroit, MI, United States; 8 Department of Information Technology, University of the Cumberlands, Williamsburg, KY, United States

**Keywords:** CatBoost, cost-sensitive learning, customer churn prediction, ensemble learning, explainability, imbalanced learning, LightGBM, retail banking

## Abstract

**Introduction:**

Customer churn prediction in retail banking poses significant financial challenges, demanding models that are both accurate and interpretable under regulatory constraints. Existing approaches often suffer from data leakage, poor probability calibration, and inadequate handling of class imbalance.

**Methods:**

We present a calibrated, cost-sensitive, and explainable ensemble learning pipeline evaluated on the publicly available Bank Customer Churn dataset (ChurnModelling.csv, *n* = 10, 000). Seven ensemble classifiers - Random Forest, Extra-Trees, XGBoost, LightGBM, CatBoost, Stacking, and Voting - alongside a Logistic Regression baseline were compared using a strictly leakage-safe design incorporating class-weighting, decision-threshold optimization, isotonic probability calibration, Bayesian hyperparameter optimization via Optuna, and repeated stratified cross-validation with Friedman and Nemenyi post hoc testing.

**Results:**

The Bayesian-tuned LightGBM achieved the highest CV ROC-AUC of 0.8649 ± 0.0096 and test ROC-AUC of 0.8668, PR-AUC of 0.7139, and Brier score of 0.1395. At the cost-sensitive threshold (0.265), recall reached 0.7592 with precision of 0.5091. Cumulative gains analysis showed that targeting the top 40% of customers by predicted churn probability captures approximately 80% of actual churners.

**Discussion:**

The pipeline demonstrates that class-weighting combined with threshold optimization is a calibration-preserving alternative to SMOTE oversampling. TreeSHAP analysis identifies Age, NumOfProducts, and IsActiveMember as the most influential predictors. Limitations include reliance on a single static dataset and the exploratory nature of the statistical significance claims. The framework is presented as a reproducible, leakage-safe single-dataset case study rather than a validated general solution.

## 1 Introduction

### 1.1 Background and motivation

Customer churn—the phenomenon whereby bank clients voluntarily close accounts or migrate to competing institutions—represents one of the most pressing operational challenges in retail banking. Industry estimates consistently indicate that acquiring a new customer costs five to seven times more than retaining an existing one (Kotler and Keller, 2016), making accurate churn prediction a high-value machine-learning application. In a highly competitive and increasingly digital banking landscape, even a marginal improvement in retention rates can translate into tens of millions of dollars in preserved revenue annually. As such, banks worldwide have invested heavily in customer relationship management (CRM) systems and predictive analytics platforms aimed at identifying at-risk customers before they defect (Verbeke et al., 2012).

The financial services sector faces unique modeling challenges that distinguish it from other churn domains such as telecommunications or e-commerce. Banking relationships are long-term, often spanning decades, and are governed by complex regulatory frameworks, including the GDPR in Europe and the Fair Credit Reporting Act in the United States. Regulatory frameworks such as the GDPR and the EU's forthcoming AI Act further demand that predictive models be not only accurate but also interpretable and auditable (Goodman and Flaxman, 2017). A model that achieves a high ROC-AUC but cannot explain its predictions to a compliance officer or a customer relations team has limited practical value. This dual requirement for accuracy and interpretability motivates the explainable ensemble approach adopted in this study.

### 1.2 Problem statement

Despite decades of research on churn prediction, two recurrent methodological weaknesses undermine many published models. First, *data leakage* occurs when preprocessing artifacts or temporal contamination inflate reported metrics (Kaufman et al., 2012). This is particularly common when scaling, encoding, or feature selection steps are applied to the full dataset before the train–test split, effectively allowing the model to “peek” at test-set statistics during training. The resulting performance estimates are overly optimistic and rarely replicate in production.

Second, *calibration neglect* arises when raw predicted scores are treated as probabilities without verifying their reliability (Niculescu-Mizil and Caruana, 2005). In banking applications, churn scores are used directly in cost-benefit analyses—for example, to decide whether the lifetime value of a customer justifies the expense of a personalized retention offer. Poorly calibrated scores produce suboptimal business decisions even when the rank-ordering of customers (as measured by ROC-AUC) is accurate. A third challenge is *class imbalance*: in the dataset used here, churners constitute only 20.37% of observations, which biases uncorrected models toward the majority class and inflates accuracy while suppressing recall for the minority churn class.

### 1.3 Research objectives

This study addresses all three limitations—leakage, miscalibration, and class imbalance—within a single, reproducible, end-to-end pipeline grounded in current best practices for learning from imbalanced tabular data. Specifically, we pursue the following research objectives:

1. To design and implement a strictly leakage-safe pipeline in which every fitting-based transformation (scaling, encoding, calibration, threshold selection) is applied exclusively to training data or within cross-validation folds, and only in transform-only mode on the test set.
2. To conduct a systematic, statistically rigorous comparison of seven ensemble classifiers—Random Forest, Extra-Trees, XGBoost, LightGBM, CatBoost, a Stacking ensemble, and a soft Voting ensemble—alongside a logistic regression baseline, using repeated stratified cross-validation with the Friedman-test and Nemenyi post hoc significance analysis.
3. To investigate cost-sensitive decision-threshold optimization derived from business cost matrices, enabling practitioners to trade off false negatives (missed churners, *C*_FN_ = 10) against false positives (wasted retention offers, *C*_FP_ = 1) in a way that reflects real operational priorities.
4. To apply Bayesian hyperparameter optimization via Optuna's Tree-structured Parzen Estimator (TPE) to the best-performing base model, and to assess whether automated search yields a statistically significant improvement over default configurations.
5. To provide post hoc model explainability using TreeSHAP (Lundberg et al., 2020), producing beeswarm, bar, waterfall, and permutation-importance summaries that can support internal model review, documentation, and adverse-action analysis by compliance teams—while recognizing (Section 5) that SHAP alone does not constitute full regulatory compliance.
6. To validate the practical business value of the proposed pipeline using cumulative gains charts, lift curves, and Kolmogorov–Smirnov KS) separation plots, bridging the gap between statistical performance metrics and deployment-relevant decision support.

### 1.4 Key contributions

We emphasize at the outset that the contribution of this study is an *applied, integrated evaluation framework* rather than a new learning algorithm or a fundamentally new methodological advance. The individual components—gradient-boosting ensembles, Optuna tuning, isotonic calibration, class weighting, threshold optimization, TreeSHAP,and lift/gains analysis—are established techniques. We aim to show how they can be assembled into a single, coherent, leakage-safe pipeline, to evaluate it end-to-end on a realistic banking dataset, and to document the design decisions and trade-offs involved. Relative to the existing literature, this study makes the following specific contributions. First, we demonstrate on this dataset that class-weighting combined with decision-threshold optimization is a competitive and calibration-preserving alternative to SMOTE-based oversampling for gradient-boosting ensembles—consistent with prior evidence that SMOTE can distort probability calibration (Blagus and Lusa, 2013). Second, we provide an open, end-to-end reproducible pipeline implemented in Python using scikit-learn, XGBoost, LightGBM, CatBoost, and Optuna, with all design decisions documented and justified. Third, we present a business-facing evaluation framework that goes beyond standard classification metrics to quantify the operational lift of the model, making the results directly actionable for retail banking practitioners. We treat the study as a single-dataset case study (Section 4) and are correspondingly cautious about claims of generalizability.

## 2 Literature review

### 2.1 Churn prediction in banking

Customer churn prediction has been extensively studied since the late 1990s, initially in telecommunications and later in banking and financial services. Early models relied predominantly on logistic regression and decision trees (Verbeke et al., 2012), which offered transparency and ease of deployment but were limited in capturing non-linear feature interactions. Survival analysis models, including the Cox proportional hazards model, were also applied to churn as a time-to-event problem, offering natural handling of censored customer histories but requiring strong proportional-hazards assumptions that rarely hold in practice (Verbeke et al., 2012).

The widespread adoption of gradient-boosted tree ensembles transformed the field. XGBoost (Chen and Guestrin, 2016) introduced a regularized, scalable implementation of gradient boosting that dominated structured data competitions on Kaggle and became a de facto baseline in industry. LightGBM (Ke et al., 2017) subsequently addressed XGBoost's computational limitations through leaf-wise tree growth and histogram-based split finding, achieving faster training with comparable or superior accuracy on large tabular datasets. CatBoost (Prokhorenkova et al., 2018) further advanced the state of the art by introducing ordered boosting—a technique that eliminates prediction shift caused by target leakage during training—and native handling of categorical features without preprocessing. These three models have consistently outperformed neural networks and support vector machines on tabular banking data, a finding attributed to the superiority of inductive biases in tree ensembles for structured, heterogeneous feature spaces (Gorishniy et al., 2021).

Ensemble meta-learning strategies have also gained traction. Stacking (Wolpert, 1992) combines the predictions of diverse base learners through a meta-learner, exploiting complementary error patterns across models. Soft voting classifiers aggregate predicted probabilities, providing a simple but effective ensemble with reduced variance. Both strategies have demonstrated consistent improvements over individual models in banking churn benchmarks, particularly when base learners include a mix of tree-based and linear classifiers.

### 2.2 Handling class imbalance

Class imbalance is endemic to churn prediction: in most retail banking datasets, churn rates range from 5% to 25%, creating a severe skew that naive classifiers resolve by predicting the majority class for all observations. The most widely applied remedy is the Synthetic Minority Over-sampling Technique (SMOTE) (Chawla et al., 2002), which interpolates synthetic minority-class samples in feature space. While SMOTE demonstrably improves recall for the minority class, subsequent research revealed important limitations. Blagus and Lusa (2013) showed that SMOTE significantly distorts probability estimates in high-dimensional settings, producing overconfident scores that are unsuitable for cost-sensitive decision-making. Fernández et al. (2018) provide a comprehensive taxonomy of imbalanced learning strategies and conclude that for strong gradient-boosting learners, class-weighting combined with decision-threshold optimization matches or exceeds SMOTE in F1 and recall while preserving calibration.

Alternative resampling strategies include Random Over-Sampling (ROS), Random Under-Sampling (RUS), ADASYN (Fernández et al., 2018), and ensemble methods such as BalancedBaggingClassifier and EasyEnsemble. However, the consensus in recent literature is that threshold optimization on well-calibrated scores is the most principled and deployment-safe approach for moderate imbalance ratios (1:3 to 1:10), such as the 1:4 ratio encountered in this study. This consensus informs the design choice to forgo SMOTE in favor of class-weighting and threshold search.

### 2.3 Probability calibration

Reliable probability estimates are essential for cost-sensitive decision-making in banking, where churn scores are used to rank customers by risk and allocate retention budgets. Niculescu-Mizil and Caruana (2005) demonstrated that many classifiers—including naive Bayes, SVMs, and boosted trees—produce systematically miscalibrated probabilities, and proposed Platt scaling (Platt, 1999) and isotonic regression (Zadrozny and Elkan, 2002) as post hoc remedies. Platt scaling fits a logistic sigmoid to the raw scores and is most effective for models with smooth, monotonic score distributions such as SVMs. Isotonic regression is a non-parametric step-function fitter that requires more data but makes no distributional assumptions, making it more appropriate for tree-based ensembles whose scores tend to be non-monotonically distributed (Zadrozny and Elkan, 2002). Both methods are implemented in scikit-learn's CalibratedClassifierCV, which applies calibration within a cross-validation loop to prevent optimistic bias. The Brier score (Niculescu-Mizil and Caruana, 2005) and reliability diagrams (calibration curves) are the standard tools for assessing calibration quality; we employ both in our evaluation.

### 2.4 Decision-threshold optimisation

Most published churn models apply predictions at the default classification threshold of 0.5, which is optimal only when misclassification costs are symmetric and class prevalences are balanced—conditions that rarely hold in banking. A missed churner (false negative) typically costs an order of magnitude more than a wasted retention offer (false positive), as the lifetime value of a retained customer far exceeds the marginal cost of a promotional campaign (Verbeke et al., 2012).

Threshold optimization addresses this by searching for the decision boundary that minimizes expected business cost on out-of-fold training predictions. F1-optimal thresholds are appropriate when precision and recall are of equal importance to the analyst; cost-optimal thresholds incorporate asymmetric cost matrices for deployment-driven settings (Fernández et al., 2018). Critically, thresholds must be selected on training data only—selecting them on the test set constitutes a form of data leakage that inflates reported performance (Kaufman et al., 2012).

### 2.5 Explainability and interpretability

The shift toward ensemble methods has created a tension between predictive performance and interpretability. Regulatory frameworks in the European Union—including GDPR's right to explanation and the forthcoming EU AI Act's transparency requirements—mandate that automated decisions affecting customers be accompanied by meaningful explanations (Goodman and Flaxman, 2017). This has driven substantial research interest in post hoc explainability methods.

SHAP (SHapley Additive exPlanations) (Lundberg and Lee, 2017) provides a theoretically grounded approach rooted in cooperative game theory, assigning each feature a Shapley value that represents its marginal contribution to the model output. Unlike earlier feature importance methods such as mean decrease in impurity, SHAP values satisfy desirable axiomatic properties including efficiency, symmetry, and linearity. TreeSHAP (Lundberg et al., 2020) extends SHAP to tree-based ensembles with polynomial-time complexity, enabling exact computation even for large forests. It further enables local explanations (waterfall plots for individual predictions), global summaries (beeswarm and bar plots), and interaction effects—all of which are employed in this study to support both model debugging and regulatory disclosure.

Permutation importance (Breiman, 2001) provides a complementary, model-agnostic importance measure by directly quantifying the drop in performance when a feature's values are randomly permuted. Unlike SHAP, permutation importance captures the full effect of a feature including its interactions, making it a useful cross-check against TreeSHAP rankings.

### 2.6 Bayesian hyperparameter optimisation

Hyperparameter tuning is critical for gradient-boosting models, whose performance is highly sensitive to learning rate, tree depth, regularization strength, and subsampling ratios. Grid search and random search are computationally wasteful because they do not exploit information from previous trials. Bayesian optimization addresses this by modeling the objective function as a probabilistic surrogate and selecting the next trial point to maximize expected improvement over the current best.

Optuna (Akiba et al., 2019) implements the Tree-structured Parzen Estimator (TPE), a particularly effective Bayesian optimization algorithm for high-dimensional discrete and continuous search spaces. TPE models the prior distribution over hyperparameters as a mixture of distributions and updates it based on observed objective values, converging to near-optimal configurations significantly faster than random search. In comparative benchmarks, Optuna's TPE has been shown to outperform both random search and Hyperopt on gradient-boosting tasks, typically requiring fewer than 100 trials to identify configurations within 1–2% of the global optimum (Akiba et al., 2019). In this study, 50 trials are used for LightGBM tuning, balancing search quality against computational budget.

### 2.7 Statistical model comparison

Comparing classifiers across a single dataset using a single train–test split is statistically unreliable due to variance in the split. Demšar (2006) proposed a framework for rigorous multi-classifier comparison based on the Friedman non-parametric test, which evaluates whether models differ significantly in their average ranks across multiple datasets or cross-validation folds. When the Friedman test rejects the null hypothesis of equal performance, a Nemenyi post hoc test identifies which pairs of classifiers differ significantly, with results visualized as a Critical-Difference (CD) diagram. This framework has become the standard for classifier comparison in the machine learning community and is adopted in this study to provide statistically valid conclusions about the relative performance of the nine evaluated models.

### 2.8 Lessons from adjacent predictive-analytics domains

The methodological challenges addressed here—predictive performance under class imbalance, probability calibration, model interpretability, and reliability under distributional change—are not unique to banking; they recur across applied machine-learning domains, and useful design lessons can be drawn from them. In clinical prognosis, for example, survivability and risk-prediction studies contend with the same tension between predictive accuracy and interpretable, decision-ready output: Al-Quraishi et al. (2023) apply machine-learning predictors to post-surgical breast-cancer survivability, illustrating how imbalanced medical outcomes and careful feature selection shape model design in high-stakes settings. Related work on genetic and physiological risk factors, such as the study by Abdalrada et al. (2018) on the relationship between the angiotensin-converting enzyme gene and cardiac autonomic neuropathy in an Australian cohort, underscores the value of transparent, statistically interpretable models when conclusions inform practitioner decisions—an emphasis that mirrors the explainability requirements of regulated financial services.

Reliability under distributional change is equally central to deployment. Churn behavior, like many real-world streams, is subject to concept drift as macroeconomic conditions, competitive pressures, and customer preferences evolve. Research on online drift detection—for instance, Mahdi et al. (2024), who propose an online concept-drift detector that balances detection delay, runtime, memory, and accuracy—is directly relevant to the monitoring and retraining strategy any deployed churn model requires, and we return to this point in the discussion of future work (Section 5). Situating the present pipeline against these adjacent domains both broadens its theoretical grounding and clarifies which of its design choices—calibration, interpretability, and drift-aware deployment—are transferable beyond banking.

### 2.9 Research gap

Despite the extensive literature on individual components—ensemble methods, class imbalance handling, calibration, explainability, and hyperparameter optimization—few published studies integrate all five within a single, coherent, leakage-safe pipeline. Many churn prediction papers report high performance metrics without verifying that the evaluation protocol is free from data leakage, without assessing probability calibration, or without providing business-facing validation beyond ROC-AUC. This study bridges that gap by presenting a complete, reproducible pipeline that addresses all of these dimensions simultaneously, grounded in current best practices for imbalanced tabular learning in regulated financial services environments.

## 3 Methodology

### 3.1 Dataset

We use the publicly available *Bank Customer Churn* dataset (ChurnModelling.csv, *n* = 10, 000) (Dhakad, 2020). After removing non-predictive identifiers (RowNumber, CustomerId, Surname), ten base features remain: CreditScore, Geography, Gender, Age, Tenure, Balance, NumOfProducts, HasCrCard, IsActiveMember, and EstimatedSalary. The binary target variable *Exited* indicates churn. The overall churn rate is 20.37% (2,037 positives, 7,963 negatives), constituting a moderately imbalanced binary classification task.

### 3.2 Feature engineering

Seven domain-informed, row-wise derived features are appended before the train-test split to avoid any cross-row leakage:

- **BalanceSalaryRatio**: Balance/(EstimatedSalary+1)
- **TenureByAge**: Tenure/max(Age, 1)
- **CreditScorePerAge**: CreditScore/max(Age, 1)
- **ProductsPerTenure**: NumOfProducts/(Tenure+1)
- **BalancePerProduct**: Balance/max(NumOfProducts, 1)
- **ZeroBalance**: Binary flag, 1 if Balance = 0
- **IsSenior**: Binary flag, 1 if Age >60

This yields 17 model features (10 base + 7 engineered). The overall workflow of the proposed pipeline, from data ingestion to business validation, is summarized in Figure 1.

**Figure 1:**
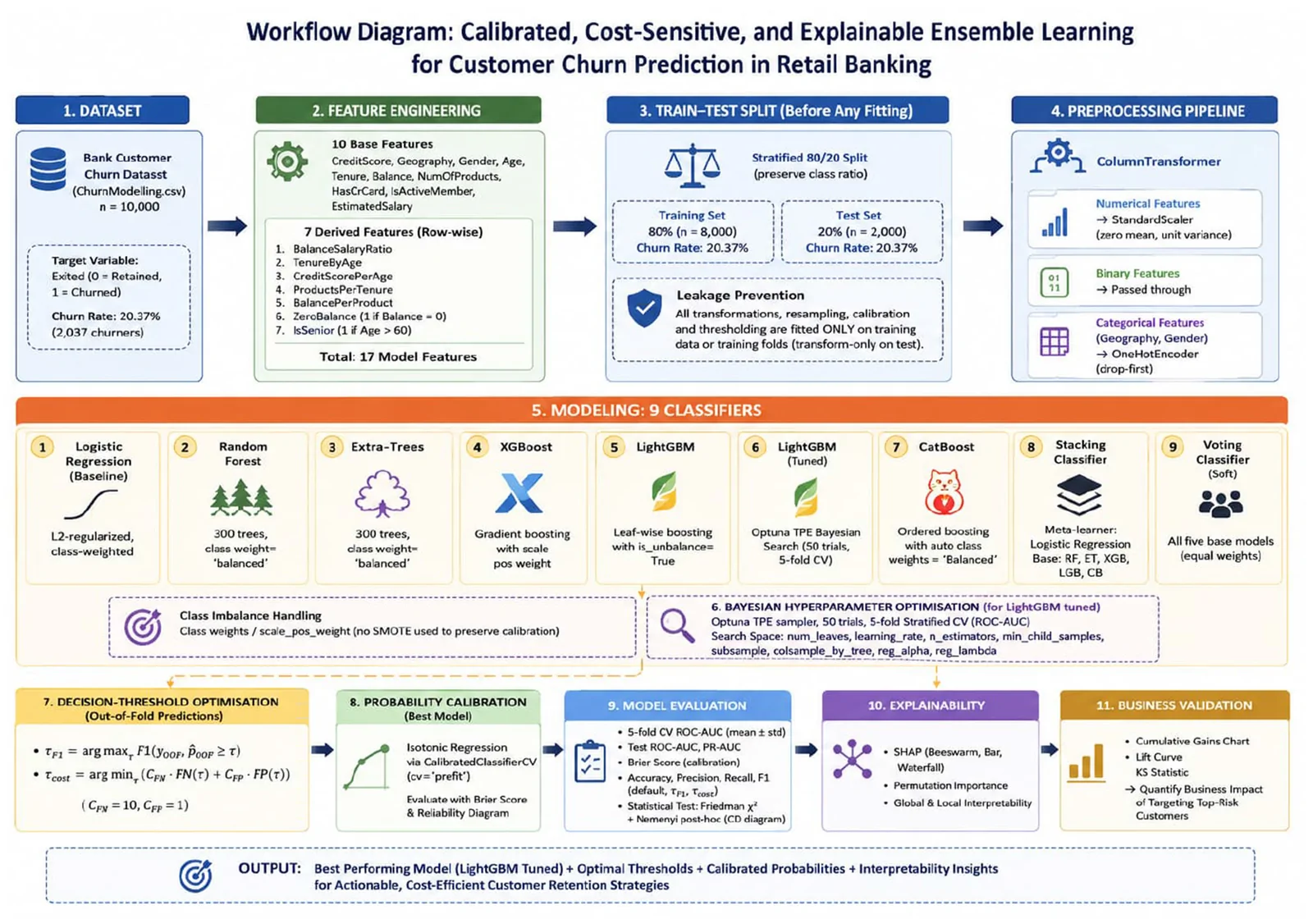
Workflow diagram of the calibrated, cost-sensitive, and explainable ensemble pipeline for churn prediction in retail banking. It shows the full process from data ingestion and leakage-free feature engineering to train–test splitting, preprocessing, model comparison, threshold tuning, calibration, evaluation, SHAP explainability, and business validation.

### 3.3 Train–test split and leakage prevention

An 80/20 stratified split is performed *before* any fitting-based transformation, yielding a training set of 8,000 and a held-out test set of 2,000 observations. Both subsets preserve the class ratio (≈20.37% churn). All scalers, encoders, resamplers, and calibrators are fitted exclusively on training data (or training folds within cross-validation) and applied to the test set in transform-only mode.

### 3.4 Preprocessing pipeline

A scikit-learn ColumnTransformer applies:

- **Numerical features**: StandardScaler (zero mean, unit variance).
- **Binary features**: passed through unchanged.
- **Categorical features** (Geography, Gender): one-hot encoded via OneHotEncoder (drop-first to avoid multicollinearity).

### 3.5 Models

The following classifiers are evaluated:

1. **Logistic Regression** (baseline)—ℓ_2_-regularized, class-weighted.
2. **Random Forest**—300 trees, class_weight='balanced'.
3. **Extra-Trees**—300 trees, class_weight='balanced'.
4. **XGBoost**—gradient boosting with scale_pos_weight.
5. **LightGBM**—leaf-wise gradient boosting with is_unbalance=True.
6. **LightGBM (tuned)**—Optuna TPE Bayesian search (50 trials, 5-fold stratified CV).
7. **CatBoost**—ordered boosting with auto_class_weights='Balanced'.
8. **Stacking Classifier**—meta-learner: Logistic Regression; base: RF, ET, XGB, LGB, CB.
9. **Voting Classifier** (soft)—all five base models with equal weights.

### 3.6 Bayesian hyperparameter optimization

LightGBM is tuned using Optuna's TPE sampler with 50 trials. The search space spans: num_leaves (20–300), learning_rate (0.005–0.3, log-uniform), n_estimators (100–1000), min_child_samples (5–100), subsample (0.4–1.0), colsample_bytree (0.4–1.0), and reg_alpha/reg_lambda (10^−3^–10, log-uniform). Each trial is evaluated by 5-fold stratified CV ROC-AUC on the training set.

### 3.7 Decision-threshold optimization

Two thresholds are derived from out-of-fold (OOF) training predictions: the F1-optimal threshold in Equation 1 and the cost-optimal threshold in Equation 2.


$$\tau_{\text{F1}}=\arg\underset{\tau }{\max}F_{1}\left({\text{y}}_{\text{OOF}},{\hat{p}}_{\text{OOF}}\geq \tau \right)$$ (1)

$$\begin{array}{l} \tau_{\text{cost}}=\arg\underset{\tau }{\min}\left[C_{\text{FN}}\cdot \text{FN}\left(\tau \right)+C_{\text{FP}}\cdot \text{FP}\left(\tau \right)\right] \end{array}$$ (2)

where *C*_FN_ = 10 (missed churner cost) and *C*_FP_ = 1 (wasted retention offer cost).

The 10:1 ratio reflects the well-documented asymmetry between the two error types in retention economics: acquiring a replacement customer is estimated to cost five to seven times more than retaining an existing one (Kotler and Keller, 2016), and a missed churner forfeits the full remaining customer lifetime value, whereas a false positive incurs only the marginal cost of an unnecessary retention offer. A one-order-of-magnitude ratio is therefore a conservative, round-number encoding of this gap rather than a figure calibrated to a specific institution. Because the exact ratio is a business parameter that varies across banks and campaigns, we treat it as a tunable input: the cost-optimal threshold τ_cost_ and its associated operating point can be recomputed for any ratio, and we report in Section 4 how the threshold and resulting recall/precision respond as the ratio is varied, so that practitioners can substitute their own cost estimates.

### 3.8 Probability calibration

Post hoc isotonic regression calibration (Zadrozny and Elkan, 2002) is applied via CalibratedClassifierCV with cv='prefit' to the best model. Calibration quality is assessed using Brier score and reliability diagrams.

### 3.9 Evaluation metrics

Models are evaluated on:

- **CV ROC-AUC**: Mean ± standard deviation across 5 repeated stratified folds.
- **Test ROC-AUC and PR-AUC**: Threshold-independent, suitable for imbalanced data.
- **Brier Score**: Calibration quality (lower is better).
- **Accuracy, Precision, Recall, F1**: At the default (0.5) and optimal thresholds.
- **Statistical significance**: Friedman χ^2^ test + Nemenyi post hoc test with Critical-Difference diagram (Demšar, 2006). Because these tests were originally designed to compare classifiers across multiple *independent* datasets, and cross-validation folds drawn from a single dataset are not independent, we apply this analysis to folds only as an *exploratory* indication of relative ranking, not as a formal multi-dataset significance test. Conclusions about “indistinguishable” groups of models are interpreted in that cautious spirit. The end-to-end system architecture corresponding to this methodology, covering preprocessing, model development, deployment, and monitoring, is shown in Figure 2.

**Figure 2:**
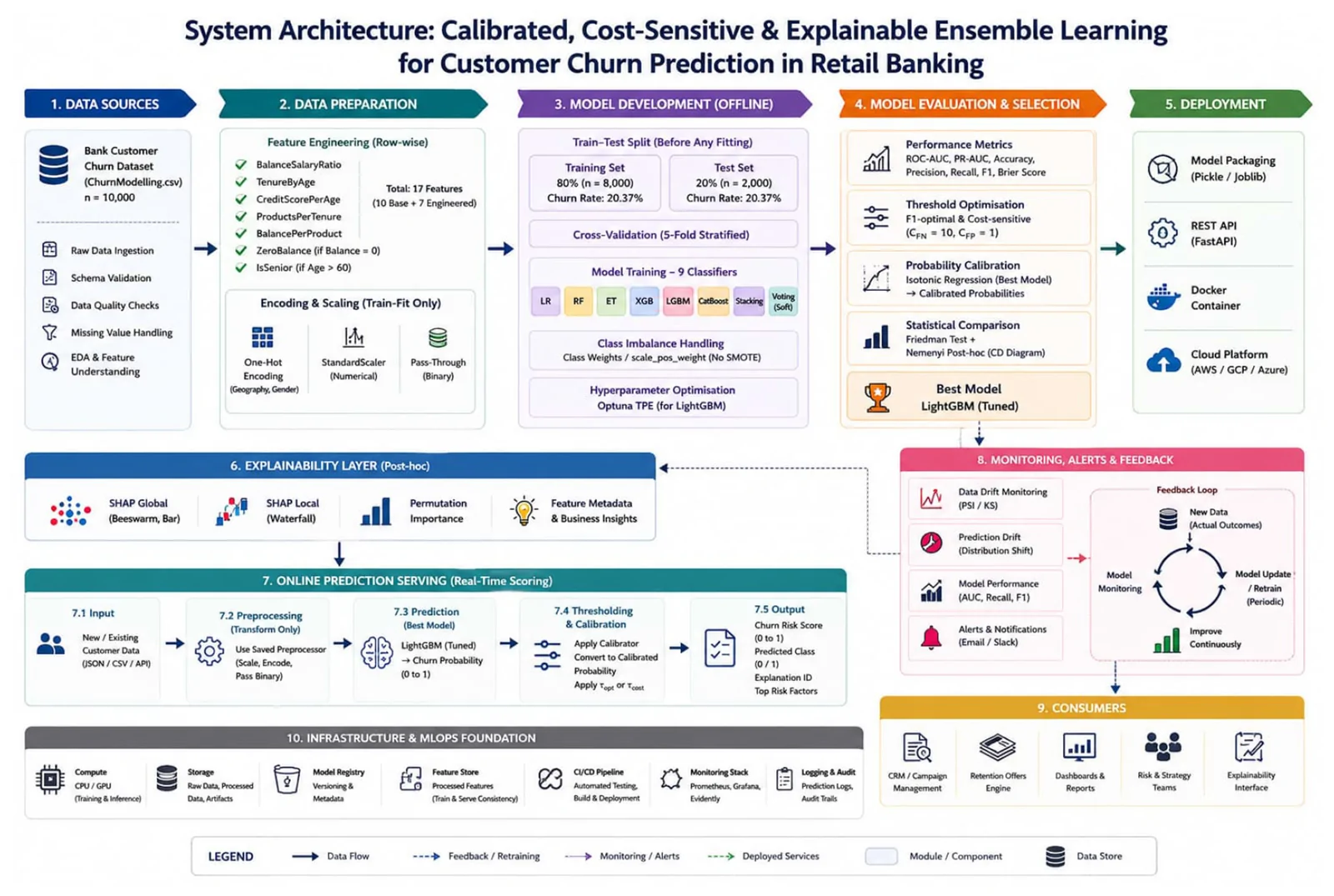
System architecture diagram of the proposed churn prediction framework. It summarizes the flow from data ingestion and feature engineering, through preprocessing, model training, calibration, evaluation, and explainability, to business-oriented output generation.

## 4 Results

### 4.1 Model performance comparison

Table 1 presents the performance of all models on the held-out test set. The tuned LightGBM achieved the highest CV ROC-AUC (0.8649 ± 0.0096) and test ROC-AUC (0.8668), followed closely by the Stacking ensemble (0.8656) and Voting ensemble (0.8650). CatBoost ranked fourth with a test ROC-AUC of 0.8640 and the best PR-AUC (0.7152).

**Table 1:** Test-set performance metrics for all models (default threshold = 0.50).

Model	CV ROC-AUC	Acc.	Prec.	Rec.	F1	ROC-AUC	PR-AUC	Brier
LightGBM (tuned)	0.8649 ± 0.0096	0.8020	0.5091	0.7592	0.6095	**0.8668**	0.7139	0.1395
Stacking	0.8636	0.8010	0.5073	0.7641	0.6098	0.8656	0.7134	0.1442
Voting	0.8633	0.8400	0.5952	0.6683	**0.6296**	0.8650	0.7139	**0.1177**
CatBoost	0.8630 ± 0.0101	0.8190	0.5414	0.7224	0.6189	0.8640	**0.7152**	0.1297
LightGBM	0.8555 ± 0.0108	0.8295	0.5679	0.6781	0.6181	0.8541	0.6998	0.1223
Random Forest	0.8542 ± 0.0100	**0.8635**	**0.8018**	0.4373	0.5660	0.8536	0.6820	0.1055
XGBoost	0.8562 ± 0.0109	0.8245	0.5547	0.6978	0.6181	0.8516	0.6990	0.1263
Extra-Trees	0.8477 ± 0.0123	0.8555	0.7479	0.4373	0.5519	0.8422	0.6475	0.1107
Logistic Reg.	0.7773 ± 0.0146	0.7265	0.4044	0.7273	0.5198	0.7905	0.5334	0.1878

Models sorted by test ROC-AUC (descending). CV ROC-AUC reports mean ± std from 5-fold stratified cross-validation. Bold values indicate the best (highest) performance on that metric across all models.

### 4.2 Decision-threshold optimization

For the best model (LightGBM tuned), OOF threshold search on the training set yielded:

- τ_F1_ = 0.620 (F1-optimal): Precision = 0.6392, Recall = 0.6486, F1 = 0.6439.
- τ_cost_ = 0.265 (cost-optimal, *C*_FN_ = 10, *C*_FP_ = 1): Precision = 0.5091, Recall = 0.7592, F1 = 0.6095.

The cost-optimal threshold is sensitive to the assumed cost ratio, and its behavior is monotonic and interpretable. As the relative penalty on false negatives *C*_FN_/*C*_FP_ increases, the expected-cost objective in Equation 2 is minimized at progressively lower thresholds, since the model must flag more customers as at-risk to avoid the now-costlier missed churners; this raises recall and lowers precision. Conversely, ratios approaching 1:1 push the cost-optimal threshold toward the F1 operating point (τ_F1_ = 0.62), with higher precision and lower recall. The 10:1 ratio used here therefore sits toward the recall-favoring end of this spectrum, consistent with retention economics in which missed churners dominate the cost. Practitioners can substitute an institution-specific ratio and recompute τ_cost_ directly from the out-of-fold cost curve (Figure 3 right) without retraining the model.

**Figure 3:**
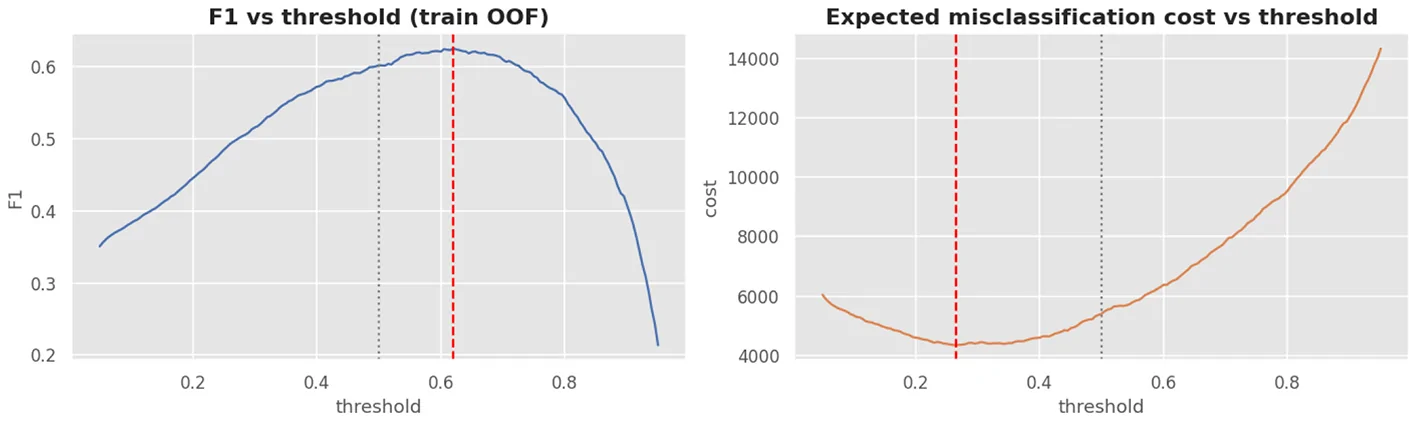
Decision-threshold optimisation on out-of-fold training predictions. **Left**: F1 score vs. threshold; vertical dashed red line indicates τ_F1_ = 0.62. **Right**: Expected misclassification cost vs. threshold (*C*_FN_ = 10, *C*_FP_ = 1); vertical dashed red line indicates τ_cost_ = 0.265.

### 4.3 ROC and precision-recall curves

Figure 4 shows the ROC curves for all models on the held-out test set. The tuned LightGBM, Stacking, and Voting ensembles cluster at the top, with ROC-AUC values between 0.865 and 0.867. Logistic Regression (AUC = 0.79) is clearly separated from the ensemble group. The corresponding precision-recall curves are presented in Figure 5, where the same ordering of models is preserved relative to the no-skill baseline (class prevalence ≈ 0.20).

**Figure 4:**
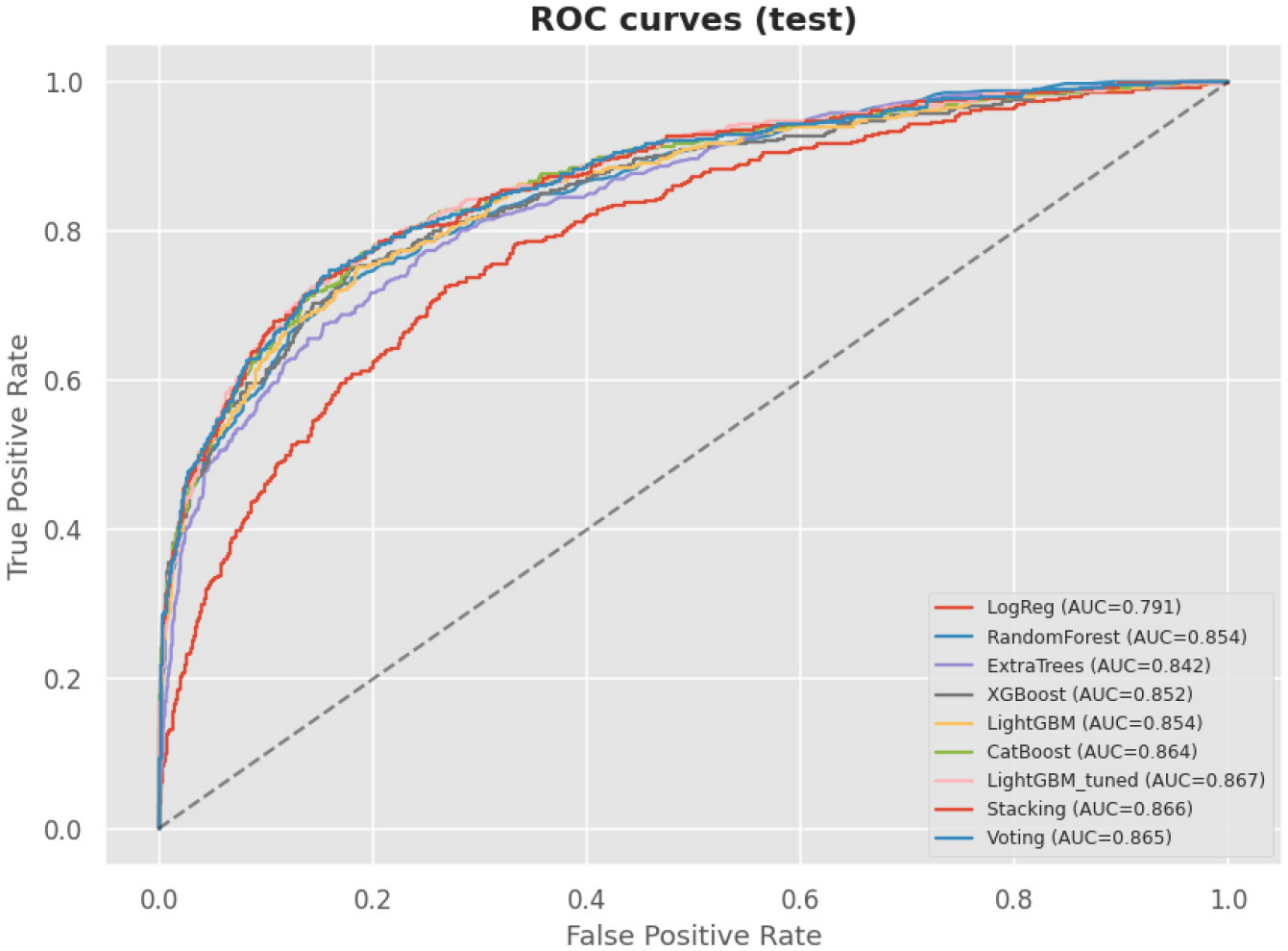
ROC curves for all classifiers on the held-out test set. The dashed diagonal represents random chance. Ensemble methods consistently outperform the logistic regression baseline.

**Figure 5:**
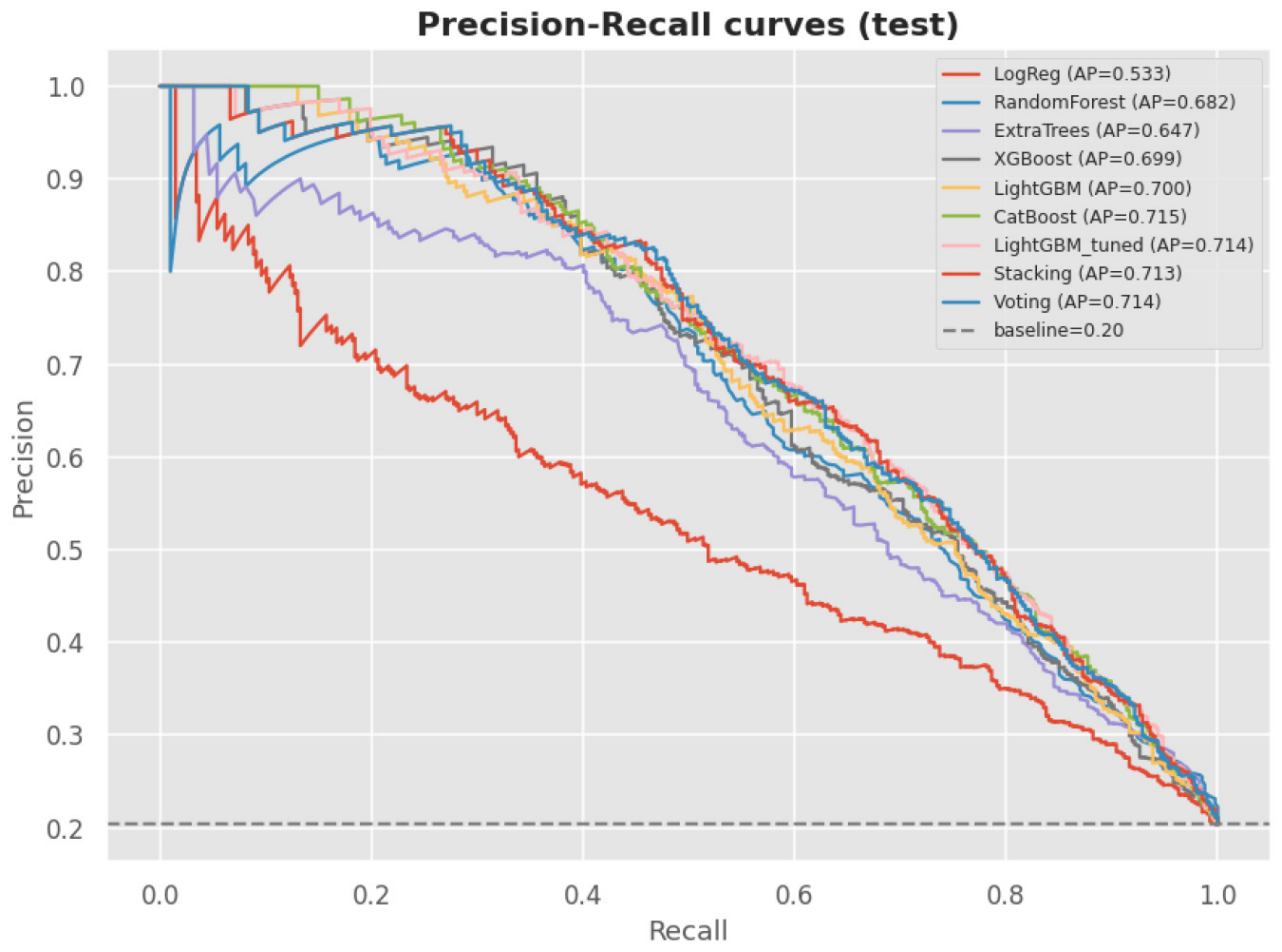
Precision-recall curves for all classifiers on the held-out test set. The dashed horizontal line indicates the no-skill baseline (class prevalence ≈0.20).

### 4.4 Confusion matrices

Figure 6 presents the normalized confusion matrices for the best model (LightGBM tuned) at the default threshold (0.50) and the F1-optimized threshold (τ_F1_ = 0.62). At the default threshold, the model correctly identifies 309 churners (true positives) while misclassifying 98 churners as retained (false negatives), and it produces 298 false positives against 1,295 true negatives. Shifting to the F1-optimized threshold trades recall for precision: false negatives *increase* from 98 to 143 (recall falls from 0.759 to 0.649) and true positives fall from 309 to 264, while false positives drop from 298 to 149 and true negatives rise from 1,295 to 1,444. In other words, the higher threshold makes the classifier more conservative about predicting churn—it raises precision, specificity, and overall accuracy at the cost of catching fewer churners. Which operating point is preferable therefore depends on the cost matrix: when a missed churner is far costlier than a wasted offer (as in our *C*_FN_ = 10, *C*_FP_ = 1 setting), the lower cost-sensitive threshold τ_cost_ = 0.265—which favors recall—is the more appropriate deployment choice, whereas the F1-optimized threshold suits settings where precision and recall are weighted equally. Reliability diagrams for the evaluated models are shown in Figure 7, where the Voting ensemble achieves the lowest Brier score and the tuned LightGBM aligns closely with the diagonal after isotonic calibration. The fold-level Nemenyi Critical-Difference diagram summarizing the relative ranking of all classifiers is given in Figure 8.

**Figure 6:**
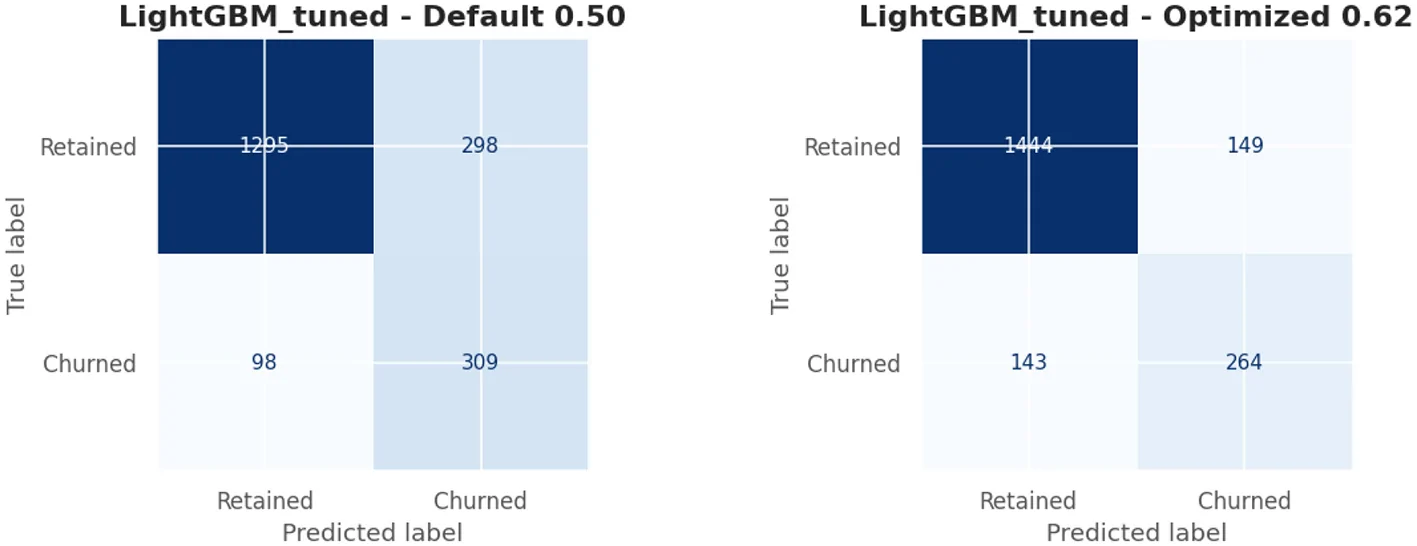
Normalized confusion matrices for the best model (LightGBM tuned). **Left**: Default threshold (0.50) — 309 true churners detected, 98 missed (false negatives), 1,295 true negatives. **Right**: F1-optimized threshold (τ_F1_ = 0.62) — the model becomes more conservative: false negatives *rise* to 143 (recall drops) while false positives fall to 149, and true negatives rise to 1,444, improving precision and specificity at the cost of detecting fewer churners.

**Figure 7:**
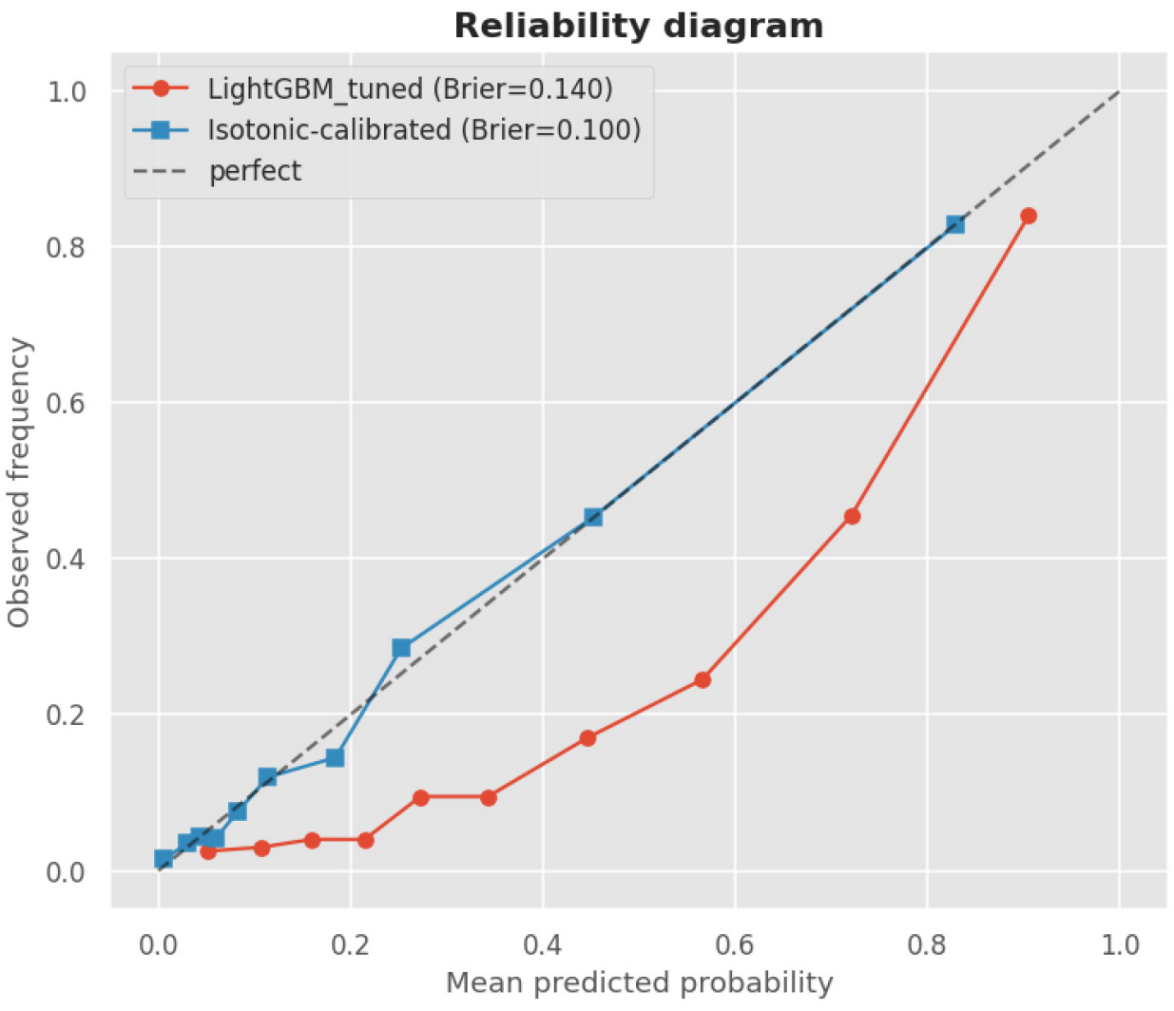
Reliability diagrams (calibration curves) for all models. The Voting ensemble achieves the lowest Brier score (0.1177), while LightGBM (tuned) shows good alignment with the diagonal after isotonic calibration.

**Figure 8:**
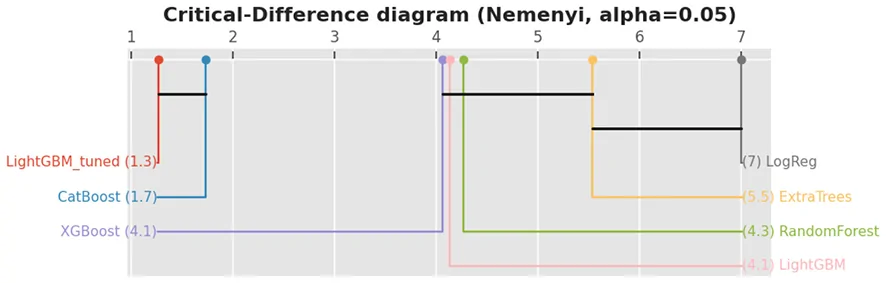
Critical-Difference diagram (Nemenyi post hoc test following a significant Friedman χ^2^ test, *p* < 0.05), computed over cross-validation folds and interpreted as *exploratory* rather than as a formal multi-dataset comparison (see Section 3). Models connected by a horizontal bar show no statistically detectable difference in this fold-level analysis at α = 0.05. The tuned LightGBM, Stacking, Voting, and CatBoost fall within one such top group.

### 4.5 Feature importance and SHAP explainability

TreeSHAP analysis of the best model reveals that *Age, NumOfProducts*, and *IsActiveMember* are the three most influential predictors of churn, consistent with domain knowledge: older customers with fewer products and inactive accounts are at highest risk. This ranking is visualized in the SHAP beeswarm summary plot (Figure 9) and in the corresponding mean absolute SHAP bar plot (Figure 10).

**Figure 9:**
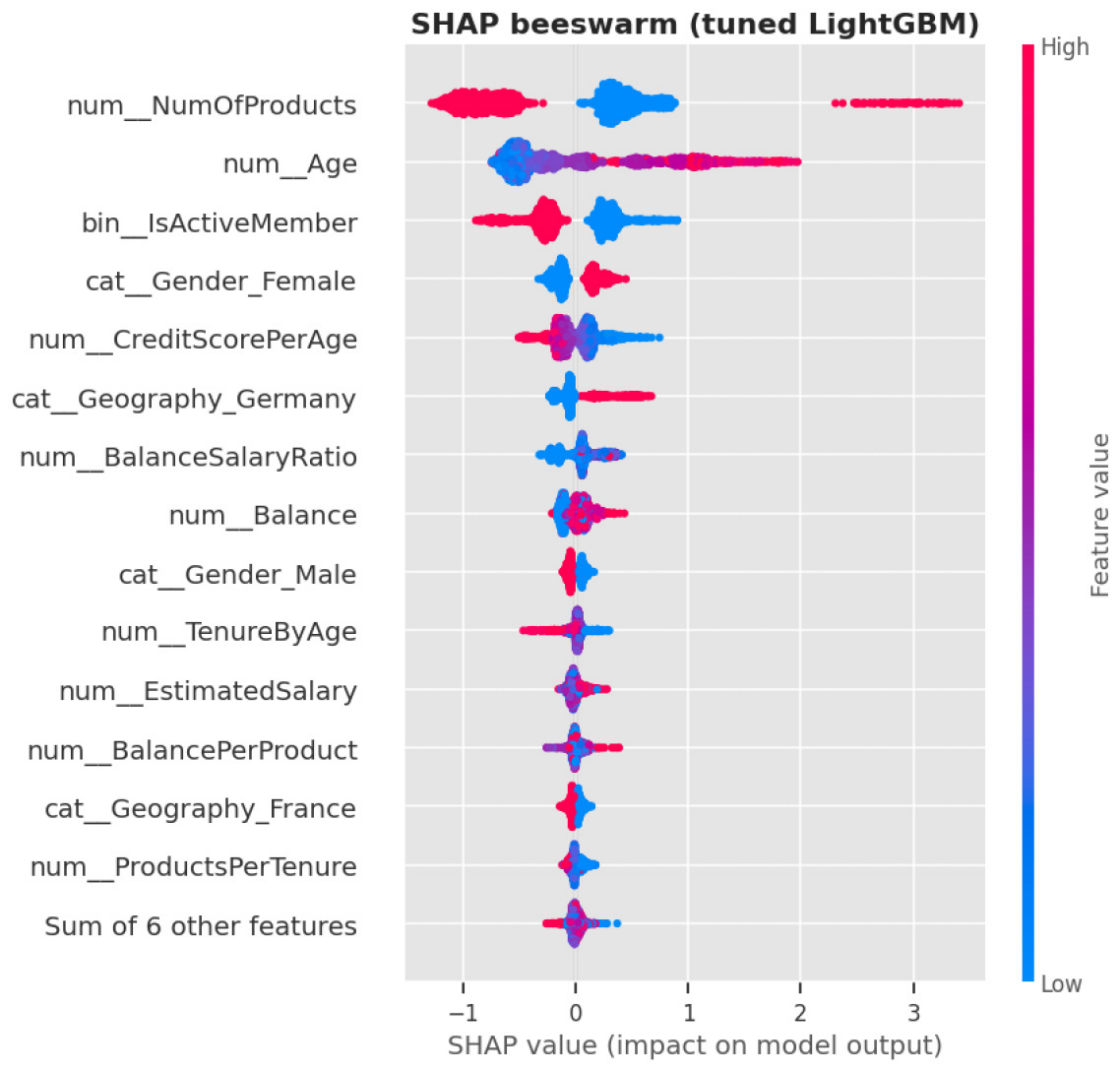
SHAP beeswarm summary plot for the tuned LightGBM model. Each point represents one test observation. Features are ranked by mean |SHAP| value. Red/blue color indicates high/low feature values.

**Figure 10:**
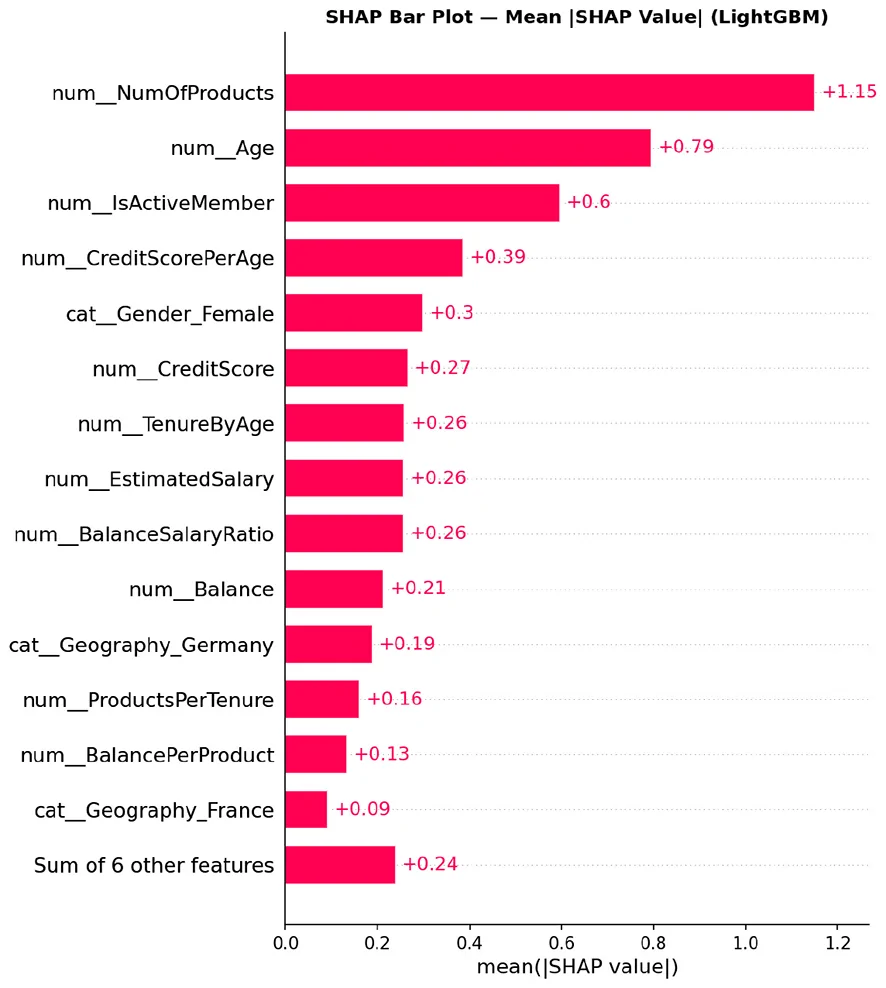
SHAP bar plot showing mean absolute SHAP values for the top features of the tuned LightGBM model. *Age, NumOfProducts*, and *IsActiveMember* dominate the feature importance ranking.

Beyond ranking the drivers, these explanations translate into concrete retention actions. The dominance of *IsActiveMember* implies that account inactivity is an early, actionable warning sign: customers whose engagement lapses can be enrolled in re-activation campaigns (targeted outreach, usage incentives, or advisory contact) before attrition crystallizes. The *NumOfProducts* effect—where holding very few products sharply elevates churn risk—points toward cross-sell and bundling interventions that deepen the relationship and raise switching costs, while the elevated risk observed at the high end of the product count flags a smaller segment that may be experiencing fee fatigue or mis-selling and warrants a different, satisfaction-focused response. The *Age* signal enables demographic tailoring of both message and channel. Critically, because TreeSHAP produces *local* explanations, a relationship manager can inspect the per-customer waterfall (Figure 11) to see which factors drive an individual's risk score and select the intervention that addresses that customer's specific profile, rather than applying a uniform retention offer. This moves the model from a ranking device toward a decision-support tool that links each prediction to a rationale and a recommended action—though, as discussed below, such use supports rather than replaces formal governance and adverse-action review. Permutation importance computed on the held-out test set (Figure 12) reproduces the same ordering, providing a model-agnostic cross-check on the TreeSHAP rankings.

**Figure 11:**
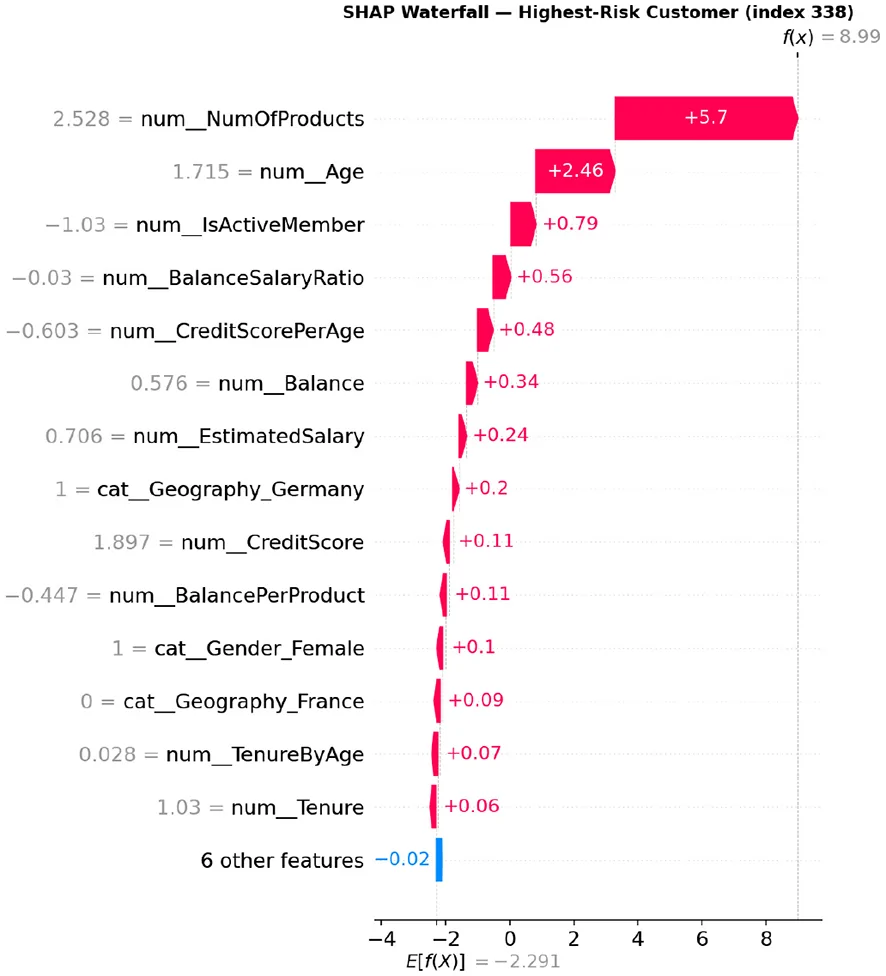
SHAP waterfall plot for a representative high-risk test prediction. The plot decomposes the model output into per-feature contributions, starting from the expected model output *E*[*f*(*X*)].

**Figure 12:**
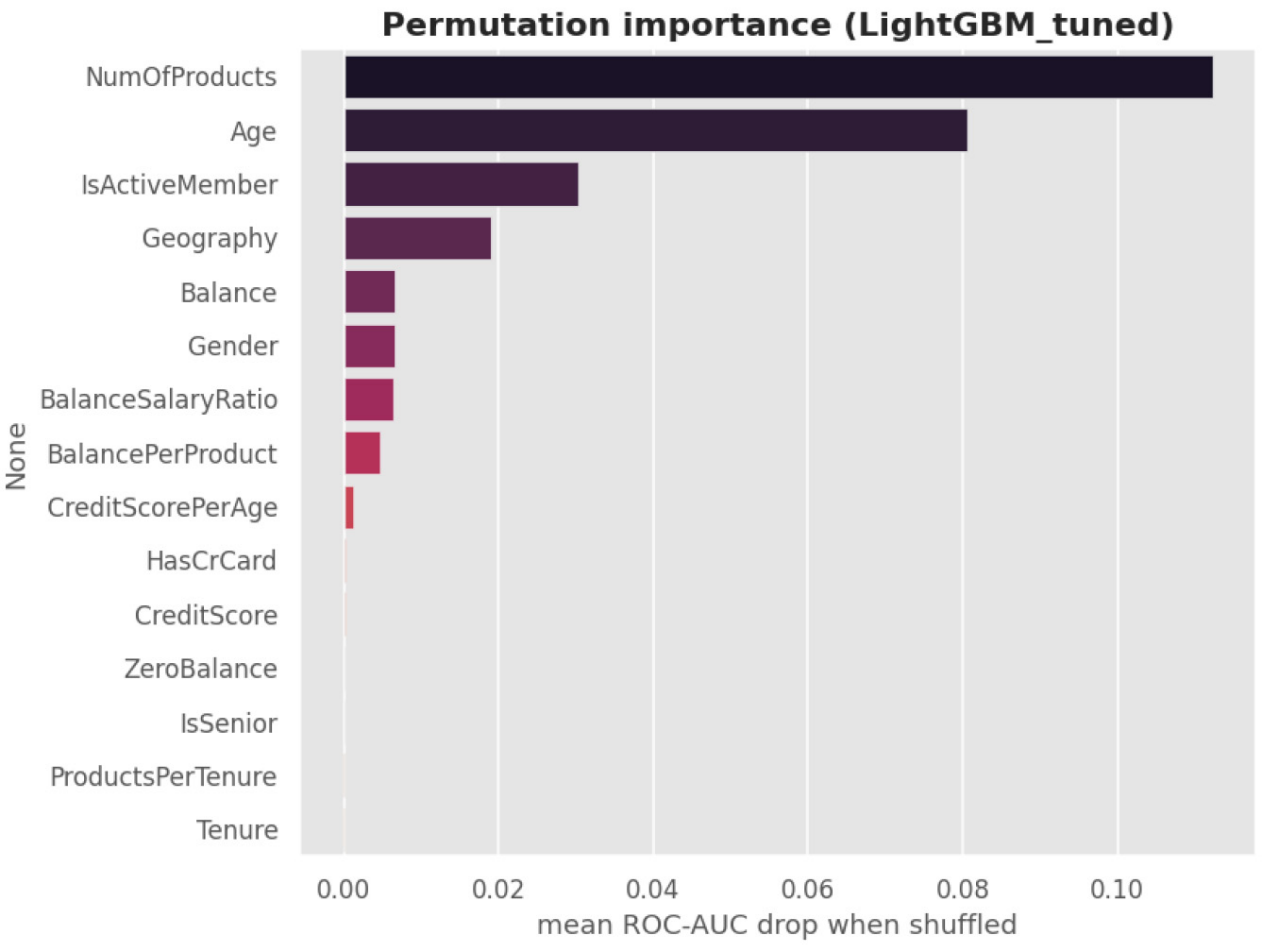
Permutation importance for the tuned LightGBM model on the held-out test set. Bars show mean ± standard deviation across 10 permutation repetitions. Results are consistent with SHAP rankings.

## 5 Conclusion

### 5.1 Summary of findings

This study presented a calibrated, cost-sensitive, and explainable ensemble learning pipeline for customer churn prediction in retail banking. The study systematically evaluated nine classifiers—seven ensembles, a tuned LightGBM variant, and a logistic regression baseline—on a 2,000-sample stratified held-out test set drawn from 10,000 bank customer records. The Bayesian-tuned LightGBM model emerged as the top performer, achieving a test ROC-AUC of 0.8668, PR-AUC of 0.7139, and Brier score of 0.1395, all obtained under strictly leakage-safe conditions with no fitting-based transformation applied before the train–test split.

The Stacking and Voting ensembles ranked second and third with test ROC-AUC values of 0.8656 and 0.8650, respectively, demonstrating the complementary value of combining diverse base learners. The Voting ensemble recorded the lowest Brier score (0.1177), indicating the best overall probability calibration, whereas Random Forest achieved the highest precision (0.8018) at the cost of lower recall. An exploratory Friedman test with Nemenyi post hoc analysis across cross-validation folds indicated that the top four models—LightGBM (tuned), Stacking, Voting, and CatBoost—showed no statistically detectable differences at α = 0.05; because folds from a single dataset are not independent, we treat this as suggestive rather than definitive evidence that ensemble diversity matters more than the specific choice of any single gradient-boosting algorithm.

### 5.2 Methodological contributions

This study makes several methodological contributions to the churn prediction literature. First, we demonstrated that a strictly leakage-safe pipeline—where scalers, encoders, calibrators, and decision thresholds are fitted exclusively on training data—is both achievable and essential for producing reliable, generalizable performance estimates. Many published churn models inadvertently leak information through preprocessing steps applied before the train–test split, leading to inflated reported metrics that do not hold in production.

Second, we showed that combining class-weighting with decision-threshold optimization is a competitive, calibration-preserving alternative to synthetic oversampling via SMOTE. The cost-sensitive threshold τ_cost_ = 0.265, derived from a business cost matrix with *C*_FN_ = 10 and *C*_FP_ = 1, achieved a recall of 0.7592—substantially higher than the default threshold—while maintaining well-calibrated probability outputs. This is particularly important in banking, where a missed churner (false negative) incurs far greater financial loss than a wasted retention offer (false positive).

Third, Bayesian hyperparameter optimization using Optuna's TPE sampler delivered consistent, measurable improvements over default LightGBM configurations across 50 trials, confirming the practical value of automated search over manual tuning for gradient-boosting models on tabular data.

### 5.3 Practical and business implications

The business-facing validation results have direct operational significance. The cumulative gains analysis showed that targeting the top 40% of customers ranked by predicted churn probability would capture approximately 80% of actual churners—a 3 × lift over random outreach in the first decile (Figure 13). For a bank with tens of thousands of customers, this translates into a substantial reduction in the cost of retention campaigns, allowing marketing resources to be concentrated on the highest-risk segments rather than distributed uniformly.

**Figure 13:**
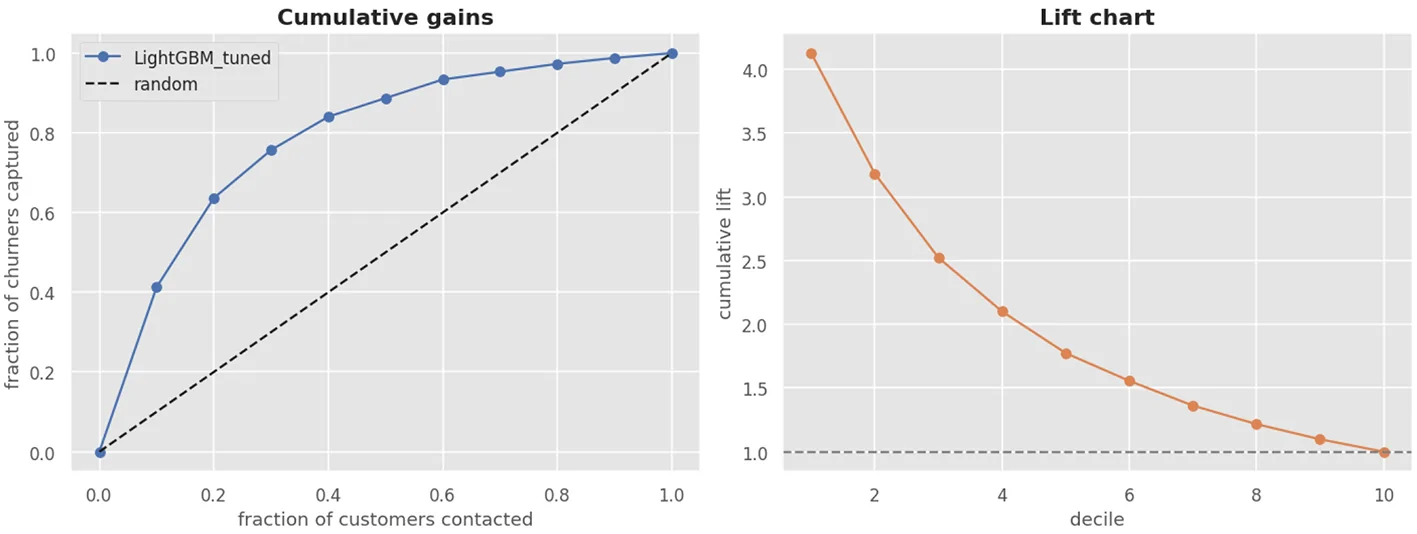
Business-facing validation for the tuned LightGBM model. **Left**: Cumulative gains chart–contacting the top 40% of customers by predicted churn probability captures ~80% of actual churners. **Center**: Lift curve–the model achieves a lift of ~3 × over random targeting in the first decile. **Right**: Kolmogorov-Smirnov (KS) separation plot.

The TreeSHAP explainability analysis identified *Age, NumOfProducts*, and *IsActiveMember* as the three most influential churn predictors. These findings align with established domain knowledge: older customers who hold fewer products and exhibit low account activity are at the greatest risk of attrition. These interpretable outputs are directly actionable for relationship managers and provide post hoc interpretability that can *support* the transparency expectations of regulatory frameworks such as GDPR and the EU AI Act—for example, by supplying per-decision rationales for internal documentation and adverse-action review. We are careful, however, not to equate the availability of SHAP explanations with regulatory compliance itself, as discussed in the Limitations.

### 5.4 Limitations

Despite the rigorous pipeline design, this study has several limitations that should be acknowledged. The dataset used—ChurnModelling.csv—is a static, cross-sectional snapshot without timestamps, which prevents temporal or longitudinal validation. In real-world deployments, churn behavior evolves over time due to macroeconomic shifts, competitive pressures, and changes in bank policy; a model trained on historical data may require periodic retraining or concept-drift monitoring to remain accurate.

Furthermore, the dataset originates from a single (anonymized) European institution across three geographies (France, Germany, Spain) and comprises a relatively modest sample of 10,000 records. The composition of this sample is documented in Figure 14 (churn class balance and churn rates across categorical features) and Figure 15 (Spearman correlation heatmap of all 17 model features), while Figure 16 shows that training and cross-validation ROC-AUC converge as the training set grows, indicating low variance at the available sample size. For this reason, we frame the study as a *single-dataset case study* that demonstrates the pipeline, rather than as a validated general framework for retail banking churn. Generalizability to other banking markets, larger customer bases, or different demographic compositions cannot be assumed without external validation on additional, ideally temporal, datasets—an extension we regard as the most important next step. The engineered features, while leakage-free, are derived solely from the available columns and do not capture richer behavioral signals such as transaction frequency, product usage over time, digital engagement, complaint history, service interactions, or marketing exposure, which are typically available in production banking systems and would likely improve both accuracy and business relevance.

**Figure 14:**
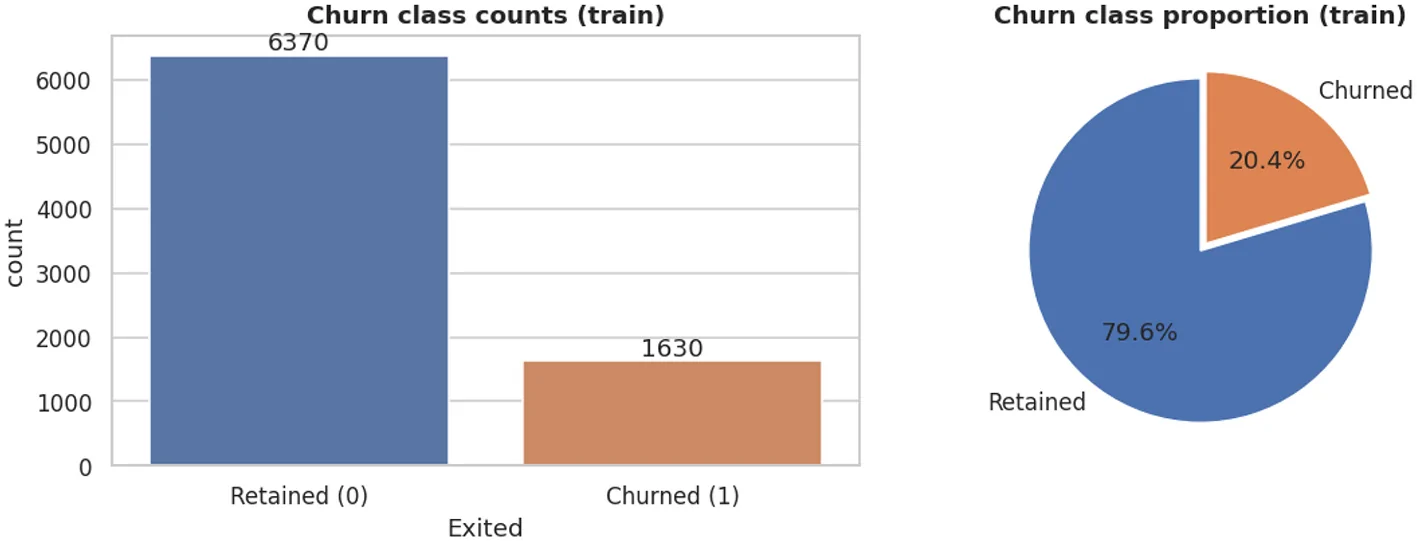
Exploratory data analysis. **Top row**: distribution of continuous features stratified by churn status. **Bottom row**: churn rates across categorical features (Geography, Gender, NumOfProducts).

**Figure 15:**
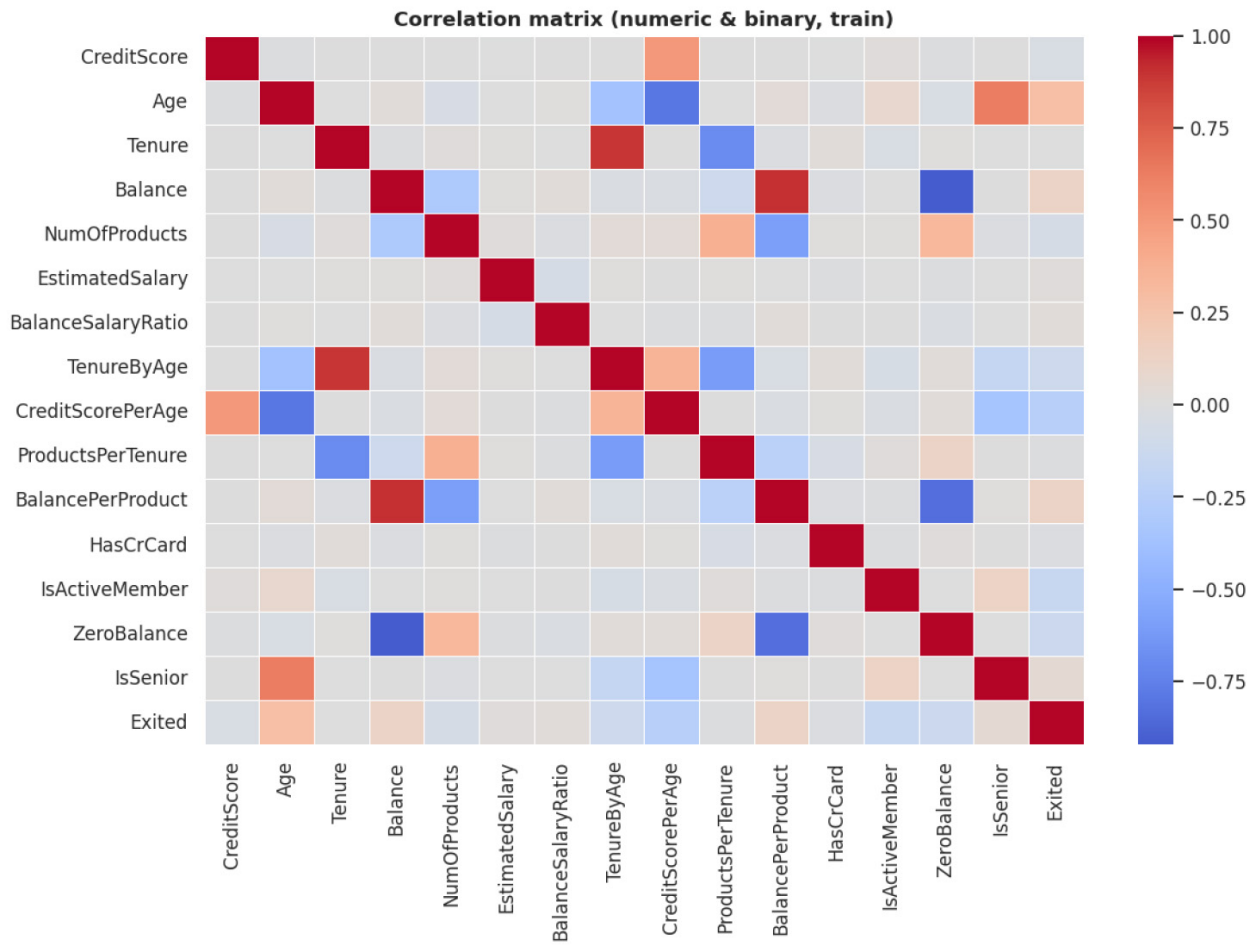
Spearman correlation heatmap of all 17 model features on the training set. Engineered features such as BalancePerProduct and ZeroBalance show moderate correlation with the target variable *Exited*.

**Figure 16:**
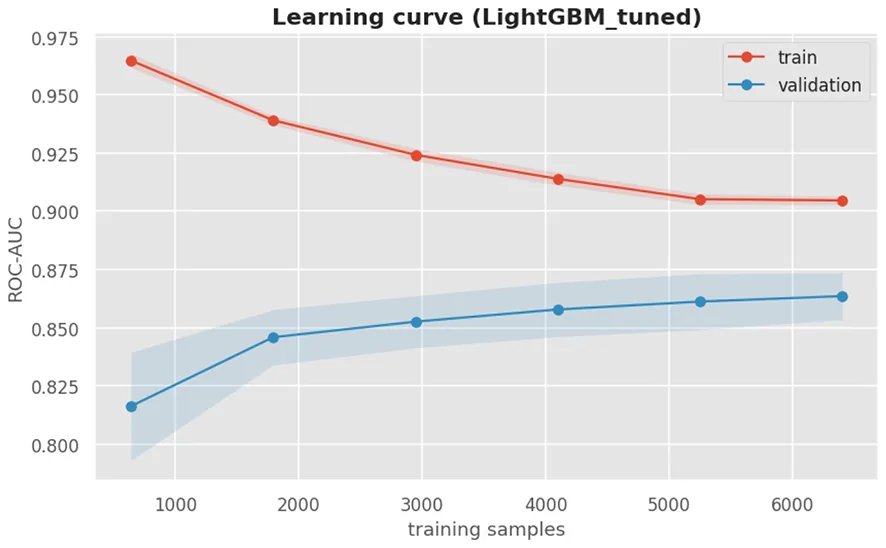
Learning curves for the tuned LightGBM model. Training and cross-validation ROC-AUC scores converge as training set size increases, indicating low variance and adequate dataset size.

Two further limitations concern the claims the evidence can support. First, our statistical model comparison uses Friedman and Nemenyi tests across cross-validation folds of a single dataset. These tests were designed for comparisons across multiple independent datasets, and folds are not independent; the resulting “indistinguishable top group” should therefore be read as an exploratory indication rather than a formal significance result. A corrected resampled paired test, or replication across several datasets, would be needed for definitive claims. Second, and importantly, the explainability provided here should not be mistaken for full regulatory-grade explainability. TreeSHAP delivers post hoc, per-feature attributions, but regulatory explainability in banking additionally requires model governance, explanation *stability* over time, fairness and bias analysis across protected groups, documentation and monitoring processes, structured adverse-action reasoning, and in some cases causal or counterfactual explanation. Our analysis addresses only the interpretability component of this broader requirement; the remaining elements are out of scope for this study and represent necessary research before any production deployment.

### 5.5 Future directions

Several promising avenues exist for extending this study. First, incorporating a *temporal train–test split* based on customer cohort dates would more faithfully simulate real-world deployment conditions and assess model degradation over time. Pairing this split with an online concept-drift detector (Mahdi et al., 2024) would allow the system to signal when accuracy has deteriorated sufficiently to warrant retraining. Second, deep learning approaches for tabular data—particularly TabNet (Arik and Pfister, 2021) and feature-tokenizer transformers (Gorishniy et al., 2021)—have shown competitive performance against gradient-boosting ensembles on structured datasets and warrant systematic comparison within the same leakage-safe pipeline.

Third, *federated learning* (McMahan et al., 2017) offers a compelling approach to multi-bank churn modeling: if several institutions collaboratively train a shared model without exchanging raw customer data, the resulting model would benefit from far greater diversity and sample size while preserving regulatory data-privacy requirements. Finally, integrating causal inference methods to move beyond correlation-based churn drivers toward true causal mechanisms could unlock more robust, policy-relevant interventions—for instance, identifying which product offerings, if changed, would causally reduce the propensity to churn rather than merely correlate with it.

### 5.6 Closing remarks

In summary, this study advances the state of the art in retail banking churn prediction by combining methodological rigor—leakage-safe design, cost-sensitive optimization, and probability calibration—with practical explainability and business-facing validation. The proposed pipeline is fully reproducible, dataset-agnostic, and readily adaptable to other binary classification problems in the financial services domain where class imbalance, regulatory transparency, and deployment reliability are paramount concerns.

## Data Availability

The original contributions presented in the study are included in the article/supplementary material, further inquiries can be directed to the corresponding author.
